# Living with males leads to female physical injury in the leaf-footed cactus bug

**DOI:** 10.1093/beheco/araf068

**Published:** 2025-06-11

**Authors:** Yichen Li, Christine W Miller

**Affiliations:** Entomology & Nematology Department, University of Florida, 1881 Natural Area Drive, Gainesville, FL 32611, USA; Entomology & Nematology Department, University of Florida, 1881 Natural Area Drive, Gainesville, FL 32611, USA

**Keywords:** female body size, female harm, male-male competition, physical injury

## Abstract

Males in many species possess sexually selected weapons that they use to fight for mating opportunities. It is well established that male-male competition can lead to physical injuries for males. However, very few studies have looked at the physical consequences for conspecific females. We hypothesized that living with males in a species with male-male competition would result in female injury. Because larger female invertebrates typically have greater reproductive output, they have higher resource value for males and can elicit aggression and fighting. Thus, we further hypothesized that larger females in this context would receive more injuries. For this study, we focused on the leaf-footed cactus bug, *Narnia femorata* (Hemiptera: Coreidae), a species of insect in which males fight using their spiny and enlarged hindlegs. In just 2 h of observation, we documented males competing with other males in 61% of 103 trials. In 43% of these 63 competitions, females were physically contacted and sometimes attacked with a kick or squeeze. We left insects in social groups for 74 h and found that females living with multiple males had a higher likelihood of obtaining injuries (26.2% of 103 trials) compared to those living only with females (9.7% of 103 trials). In addition, larger females were more likely to be injured compared to smaller females. Our study highlights the harm that females can experience in species with male-male competition.

## Teaser

Competition between males frequently leads to male injuries. But the extent to which male-male competition results in female injuries is largely unknown. In the leaf-footed cactus bug, we found that aggressive males not only attack rival males, but also harm nearby females, especially larger females. Female injuries were varied, ranging from punctures through their skeleton, to broken wings, and missing appendages.

## Introduction

Males in tens of thousands of species have evolved elaborated sexually selected weapons, such as tusks, antlers, spines, and horns (Emlen 2008). Males use these weapons in contests with other males over reproductive opportunities. Combat between males can be vicious and costly. Severe injuries and even death after fighting in males are not uncommon (Bean and Cook 2001; Sneddon et al. 2003; Kelly 2006; Valero et al. 2006; Emlen 2008; Lane and McCullough 2025). However, few empirical studies have investigated whether male-male competition leads to female injury.

There are multiple non-exclusive reasons to expect that females may be directly or indirectly attacked and harmed during male-male competition. First, female harm caused by male conspecifics is not uncommon (Clutton-Brock and Parker 1995), even in species without aggressive male-male competition (Crudgington and Siva-Jothy 2000; Blanckenhorn et al. 2002; Kamimura et al. 2016). Consequences for females may be varied, including reduced reproductive output (Rossi et al. 2010; Li et al. 2015). Second, male sexually selected traits and fighting behaviors can serve as important cues of male quality for female mate choice (Berglund et al. 1996). Thus, females may be drawn to closely inspect male contests, thus increasing their risk of injury. Third, male-male competition may become heightened when more females, and especially fertile, fecund females, are present (Emlen and Oring 1977; López and Martín 2002; Procter et al. 2012; Álvarez et al. 2017). Finally, male contests can continue during mating. Rival males sometimes attack mating pairs (Jacob 1955; Edvardsson and Tregenza 2005). These interactions suggest that the presence of competing males may pose risks to females, an idea indicated in early works (Daly 1978). However, most studies on costs of mating for females focused on sexual coercion (Le Boeuf and Mesnick 1991; Clutton-Brock and Parker 1995; Lange et al. 2013) with limited research on male-male competition. Therefore, much less is known about the extent of harm females may experience when being around competing males.

Our primary goal was to investigate the effect of living with males on female injury in a species with male-male competition. We focused on a member of the leaf-footed bug superfamily, Coreoidea, where males of many species fight end-to-end using their spiny hindlegs over access to territories and reproductive females (Miller et al. 2024). Though female-female competition has been documented as a way to secure preferred males in other species (Chuard et al. 2016), females have not been witnessed fighting over 13 yr of field and laboratory research in *Narnia femorata* (Hemiptera: Coreidae). However, males readily use their elaborated hindlegs to kick and wrestle over access to cactus territories that females visit for feeding and egg-laying (Procter et al. 2012; Nolen et al. 2017). Their mating system can be described as resource defense polygyny (Emlen and Oring 1977). Varied contest behaviors include displaying legs, charging at opponents, mounting opponents, kicking, and squeezing each other end-to-end (Nolen et al. 2017). During the hindleg squeeze, males press sharp hindleg spines into the body of their opponent, and this behavior can lead to physical injuries ranging from scratches, to punctures, to even loss of appendages (Raina et al. *unpublished manuscript*).

We predicted that female *N. femorata* residing with multiple males would be more likely to get injured compared to females living only with other females. Our rationale for this prediction was based on multiple observations of males disrupting mating by attacking male-female pairs on host plant territories. We also predicted that larger females would be more likely to be harmed because male *N. femorata* favor larger females (Gillespie et al. 2014). Indeed, large females commonly produce more offspring than small females (Miller et al. 2013), which may benefit male paternity and incite more aggressive contests.

In addition to our primary goal, we pursued three secondary objectives in this study, all chosen to provide context and understanding of the attacks that enmesh females and the consequences of such attacks. To this end we (1) conducted behavioral observations of insect interactions in social groups, (2) categorized the types of injuries that females sustained, and (3) noted any consequences for short-term female offspring production.

Our behavioral observations were conducted during the first 2 h of our 74-hour experiment. Our major aim of these observations was to document the frequency of physical attacks that involved females relative to male attacks in general. We also expected females that mated more and those that mated with multiple males would be more likely to become injured. Thus, we documented whether females copulated. We further examined whether the presence of (1) male-male competition and (2) squeeze attacks on females in these first 2 h would predict the frequency of female injury documented at the end of the study.

After the 74-hour experiment, we carefully inspected female bodies to record and categorize any injuries received. Finally, we examined one potential consequence of such injuries—negative effects on offspring production during the study period. Previous work has shown that injuries in insects can cause costly immune responses (Schmid-Hempe 2005), which could result in less energy allocated to reproduction (Schwenke et al. 2016). Thus, we predicted that females with physical injuries would produce fewer hatchlings compared with females with no injuries.

## Materials and methods

### Insect rearing

We used *Narnia femorata* (Stål, 1892) (Hemiptera: Coreidae) from a lab colony established in 2022 and supplemented in 2023 with wild-caught individuals from Live Oak, Florida (30.2642 N°, 83.1768° W). All the insects were kept in a rearing room with temperatures ranging from 26 to 28 °C. We reared nymphs in plastic deli cups capped with mesh lids. We kept the density at 5 to 20 siblings per cup since nymphs are usually found in groups in nature (Allen and Miller 2020). We also provided each cup with a prickly pear cactus pad (*Opuntia mesacantha ssp. lata*) planted in soil with an adjacent cactus fruit. We monitored the quality of cacti and cactus fruit throughout the project and moved bugs to a new cup if cactus quality declined.

When the nymphs emerged into their penultimate instar, we separated each one into an individual deli cup with a planted cactus pad and a fruit. They were kept in deli cups by themselves to minimize social interactions and to ensure the females remained unmated. Once an individual emerged as an adult, we assigned it a unique code and marked it using a non-toxic paint pen. All adults were marked at least 1 d before the experiments started to minimize any effects of this disruption during the experimental period.

### Treatments

There were two treatments, each with 103 replicates, a 1) female-only treatment, and a 2) mixed-sex treatment. Each group of the female-only treatment consisted of one focal female and three background females; each group of the mixed-sex treatment consisted of one focal female and three males. For the mixed-sex treatment, we mimicked an operational sex ratio where more males are seeking mating than are females, a situation likely common in nature (Kokko et al. 2014). The distribution of these insects in the wild suggests the formation of hidden leks (*sensu*Fletcher and Miller 2006), and it is also not uncommon in the leaf-footed bug superfamily to find multiple males on a host plant as they vie for a coveted territory (Miyatake 1995). Not considering reproductive state or social groupings, the primary sex ratio of *N. femorata* in the wild is typically 1:1 (Cirino and Miller 2017). Females mate multiply, and work on other hemipteran insects suggests that last-male sperm precedence is common (Parker 1970; Gwynne 1984).

We assigned insects randomly to a treatment group. Some unmated females were used as background females in two different replicates of the female-only treatment. Each male was used in the mixed-sex treatment up to five times. We visually inspected each focal insect before assigning it to a treatment and did not include those with obvious physical damage.

### Experimental design

There were two parts of the experiment: a two-hour observation period followed by a 72-hour non-observation period. During the observation period, we conducted behavioral trials at approximately 29 °C, noting their fighting and copulating behaviors. Before each trial, we allowed selected insects to acclimate to the environment in mesh-covered plastic cups alone for around 30 to 60 min before putting them into their assigned group. All insects were gently moved into their assigned deli cup with a cactus and a ripened fruit. We also placed pine needles into the container, which females used as their main egg-laying substrate.

Each observation period lasted for 2 h, and we watched up to 9 groups at a time. It was feasible to track behaviors for multiple groups at the same time because these insects interact only intermittently and sometimes appear to ignore each other completely (Nolen et al. 2017). We defined male-male competition (or male contest) as one or more behaviors that include the following: leg displaying, charging, mounting, kicking, or squeezing. We defined these behaviors as in the ethogram (Table S1) modified from Nolen et al. (2017). We conducted observation on these behaviors for both mixed-sex and female-only treatments. The squeeze attack, where males squeeze each other using their spiny hindlegs, and often end-to-end, is part of male-male competition, and is the behavior most likely to cause injury because of the sharp spines and the force generated as the tibia and femur of one or both legs are squeezed together. In fact, squeezing can be felt as a pinch and even can draw blood when some leaf-footed bugs are handled by humans (Miller et al. 2024). Thus, we paid special attention to hindleg squeezes in one of our statistical analyses. Copulation was noted when a male mounted a female, initiated genital contact, and females opened their genital plates to allow copulation. Copulation was only considered successful when a male subsequently dismounted and rotated outward to form an obtuse angle with the genitalia still connected. We documented the number of copulating pairs that were contacted by rivals and whether a kick or squeeze occurred.

After the two-hour observation period, we moved all the groups back to the 26 to 28 °C rearing room for another 72 h. We provided this extended period to allow insects to interact further and females in the mixed-sex treatment to lay eggs so that we could quantify their reproductive output. Females in the female-only treatment were always unmated. After this non-observation period, we froze the focal female in each group for both treatments for later measurement and bodily injury scoring.

### Injury scoring

We scored female injury blind to treatment using a Leica M165C stereo microscope. We used ImageJ v.1.54d (Schneider et al. 2012) to measure pronotum width as a proxy for body size (Allen and Miller 2017). We counted and categorized observed physical injuries as follows: (1) antennal injury, including broken antennae and missing setae; (2) leg injury, including broken spines, melanization, exoskeletal punctures, and missing legs; and (3) wing injury, including wing tears and wing fragmentation. We noted evidence of melanization because it suggests wound healing in insects (Gillespie et al. 1997).

### Offspring production

We assessed one potential negative consequence of injury—a decrease in the production of offspring. We only collected these reproductive output measurements for the mixed-sex treatment groups because females remained unmated in the female-only groups. We assessed the number of live offspring produced from eggs laid during the 74-hour experimental period. We counted the number of hatchlings based on the number of opened pseudo-opercula on Day 14 after the experiment. Hatchling counts were collected by two people, blind to the other’s counts, and we used the average of these two numbers.

### Statistical analyses

All statistical analyses were conducted using IBM SPSS v.28. We first tested the consequence of the mixed-sex versus the female-only treatment on female injury after the 74-hour experimental period. We used a Generalized Linear Model (GLM) assuming a binomial distribution with female injury (presence or absence) as a binary response variable. The explanatory variable was social group treatment (mixed-sex or female-only), which was considered as the main effect. We used female body size as a continuous covariate. In this and in all models, we first included two-way interaction. We a priori decided to use the stepwise elimination procedure (Hardy and Field 1998), where non-significant interactions are removed. We conservatively decided a priori that only interactions with *p *> 0.10 would be removed.

Next, we examined whether an observed aggressive squeeze attack on a female was predictive of female injury at the end of the 74 h. We constructed a binomial GLM with female injury (presence or absence) as the binary response variable. Our model tested if an observed squeeze attack on a female predicted female injury at the end of the study. This GLM included female body size as a continuous covariate.

Finally, we examined the effects of female body size and injury on offspring production. The hatchling count data was over dispersed, thus we ran a GLM fit to the negative binomial distribution. Our model included the number of hatchlings as the response variable and both female injury (presence or absence) and female body size as explanatory variables.

## Results

**Fig. 1.**
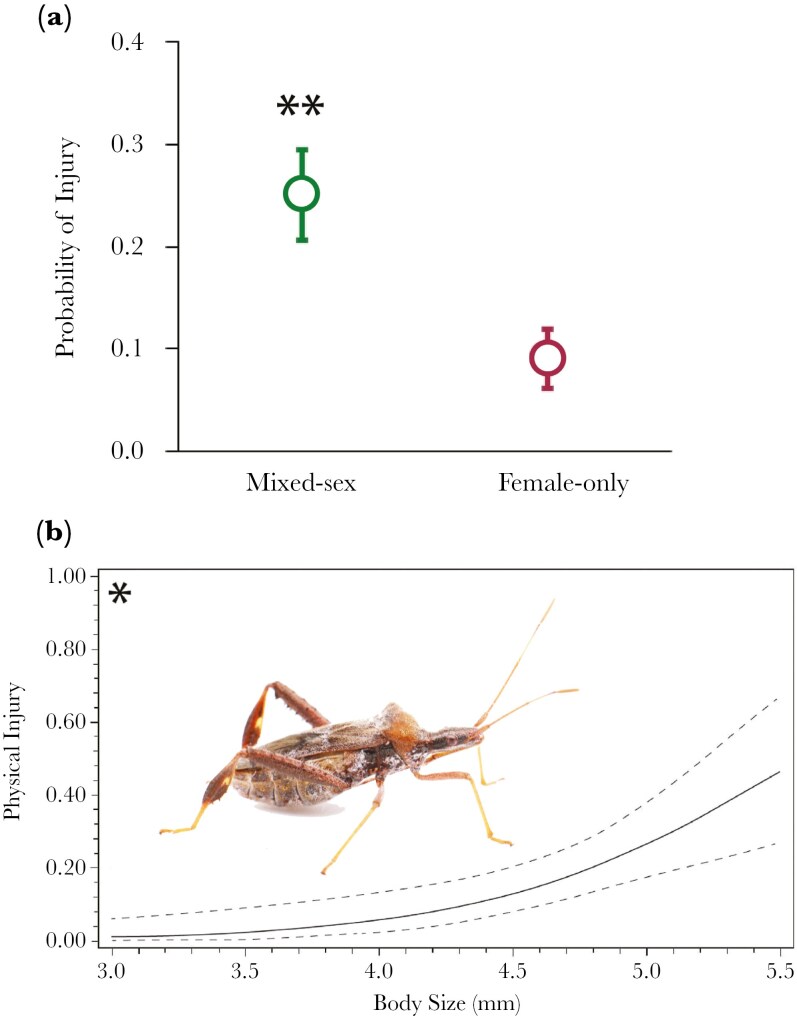
Females in mixed-sex social group and larger females are more likely to be injured. (A) Probability of getting injury for female in mixed-sex and female-only treatments. Females in the mixed-sex treatment have 26.2% chance of getting injured compared to 9.7% in the female-only treatment (** indicates *p *< 0.01). Bars represent ± standard errors. (B) Cubic spline (Schluter 1988) based on raw data showing probability of getting injury for female with different body sizes. Larger females are more likely to get injured compared to smaller females (* indicates *p *< 0.05). Dashed lines are ± 1 standard error of predicted values generated from 50 bootstrap replicates. Photo by Christine W. Miller.

Female body size did not significantly differ between treatments (GLM: Wald χ2 = 1.945, d.f. = 1, *p = *0.163). As expected, we found that females in the mixed-sex treatment were almost three times more likely to show signs of injury (GLM: Wald χ2 = 8.108, d.f. = 1, *p = *0.004; Fig. 1A). After just the 74 h that insects were kept together, 37 females across both treatments showed signs of injury out of the 206 females. Twenty-seven of these injured females were in the mixed-sex treatment group (26.2% of n = 103), while only 10 were in the female-only treatment (9.7% of n = 103). We also found that larger females were more likely to be injured (GLM: Wald χ2 = 4.063, d.f. = 1, *p = *0.044; Fig. 1B).

### Behavioral observations

We observed fighting and copulation over the first 2 h of the experimental period. We did not observe any female-female aggression in the female-only treatment. They sometimes walked over each other, but did not show aggressive behaviors toward each other. Of the 103 mixed-sex groups, 63 (61%) of these groups had two or more males interact with a leg display, charge, mount, kick, or squeeze, which we considered as signs of aggression. Some incidences of mild male-male competition only included a leg display, charge, or mount, while other include a kick or squeeze. We noticed that occasionally males engaged in squeezing from the top of deli cup and dropped to the bottom during the process. Sometimes as they fell, they incidentally hit the female. Males in some groups were especially competitive, and these males were more likely to chase females in an aggressive manner. As a results, females in these groups were more likely to fly and avoid males.

**Fig. 2.**
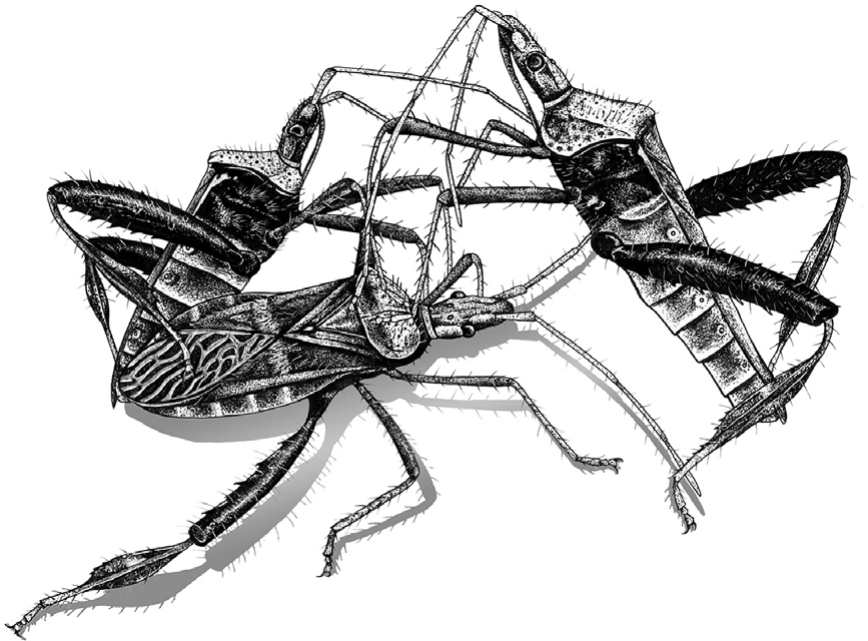
A rival male interrupting a mating pair in *Narnia femorata*. The mating pair (left) includes a female (foreground) and a male (background). The rival male (right) is mounting the mating pair while the mating male is swinging around to face the rival. The illustration (by David J. Tuss) depicts an observed contest, whereby the rival subsequently attacked the mating pair using his hindlegs to squeeze both the mating male and the female. In the process, the mating ended. See also Videos S1 & S2.

As expected, mating pairs were commonly contacted and sometimes attacked by a rival male (Fig. 2; Video S2). We recorded that 94 out of 103 (91%) mixed-sex treatment groups had one or more copulation. In 24 of those cases, a female mated with multiple males during the two-hour period. We recorded 63 separate copulations during the two-hour observation window, and in 31 cases, the mating pair was contacted by a rival. Of the 31 times rival males contacted mating pairs, 10 contacted without observable combat (32%) while 21 (68%) contacted with combat that included at least one male contest behavior. Among the 21 mating pairs that were contacted with combat, 9 received at least one squeeze with the hindlegs. When a rival male attacked a mating pair, he typically started by approaching and mounting the female, and then a fight was initiated by either the rival or the mating male. One male usually raised a hindleg or charged the other, and then the fight escalated where they squeezed each other with the female underneath them until one male left or the female ran away. There were also times where the rival males took over the mating opportunity without any fight.

While we expected males to attack mating pairs, we were surprised that five females that were not engaged in copulation experienced a squeeze attack from a male. One female was squeezed when mating and, later, alone. Another female experienced a squeeze on her left antenna and had her whole body lifted for several seconds due to the male squeeze. She remained lifted and held herself still until the male released her antenna (Video S1). When females were attacked alone, they usually moved away quickly. When they were attacked during mating, they needed to disconnect with their mate before running away.

Our observations amounted to 2.7% of the 74 h insects spent together. We did not find evidence that the presence of squeeze attack on a female during this observation period was predictive of female injury assessed at the end of the study (GLM: Wald χ2 = 0.526, d.f. = 1, *p = *0.468). Although we documented 13 instances of females receiving a hindleg squeeze from a male, only four of these females were clearly injured by the end of the study (31%).

### Injury types

**Fig. 3.**
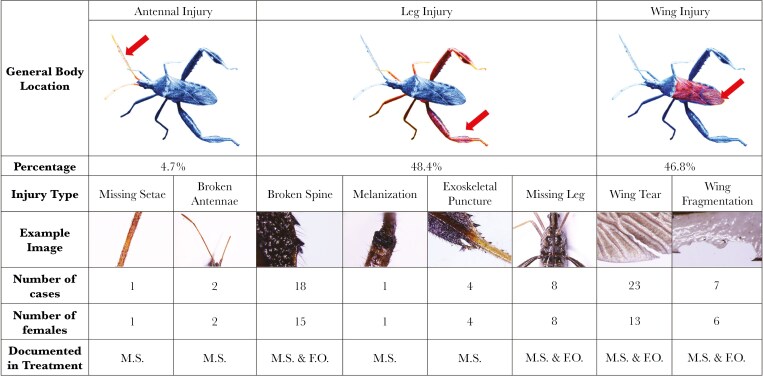
Types of observed physical injuries documented in female *Narnia femorata* across treatments. M.S.= Mixed-sex treatment; F.O.= Female-only treatment. Insect photo by Christine W. Miller. Injury photos by Yichen Li.

We documented injuries in both the mixed-sex groups (26.2% of females) and the female-only groups (9.7% of females). Injuries were varied in type (Fig. 3) and scale (see Table S2). Of the 37 injured females 43.2% exhibited broken or torn wings, 40.5% had broken spines, and 21.6% had missing legs. We recorded a total of 64 injuries across the 37 females. Of the injuries that were not to the wings, 29.7% were on the hindlegs, 18.8% were on the other legs. Extreme injuries documented including missing legs, large fragments of the wings missing, broken antennae, and punctures through the cuticle. Four injury types were not commonly observed from injured females in the female-only groups. These included broken antennae, missing setae (only one case), melanization (only one case), and puncture wounds to the exoskeleton.

### Offspring production

The 103 focal females in the mixed-sex treatment laid 827 eggs (mean ± SD: 8.03 ± 6.97, range: 0 to 27) in 74 h and 694 of these eggs later hatched (84%). As expected, larger females produced more hatchlings (GLM: Wald χ2 = 9.929, d.f. = 1, *p = *0.002; Figure S1). Physical injury was not associated with a decrease in the number of hatchlings produced during the 74-hour period (GLM: Wald χ2 = 0.024, d.f. = 1, *p = *0.876; Figure S2).

## Discussion

We investigated whether living with multiple males is costly for females in the leaf-footed cactus bug, *Narnia femorata*. This insect species has enlarged hindlegs and males use these legs as weapons for male-male competition. We experimentally grouped each focal female with either three males or three other females to test whether living in a mixed-sex group would lead to more physical injuries. First, we found that females living with multiple males had nearly three times the chance of being injured compared to females living with only females (Fig. 1A). Additionally, larger females were more likely to get injured compared to smaller ones (Fig. 1B).

Our finding supports the hypothesis that females living with males are more likely to be injured. This is consistent with other studies. As documented in water strider *Gerris buenoi* (Rowe et al. 1994) and dung fly *Sepsis cynipsea* (Mühlhäuser and Blanckenhorn 2002), being around greater number of males can result in higher incidence of mating harassment and increase the rate of injury. In *N. femorata*, males often fight intensely when females are present (Procter et al. 2012), and they sometimes attack mating pairs (Fig. 2; Video S2). We learned here that male *N. femorata* may also direct their attack to nearby females, even when females are not mating or even near other males. However, for over hundreds of hours of observation, we found that such attack towards females is most common when rival males are in close physical proximity.

Female harm from male-male competition has been seen in a number of vertebrates (Daly 1978). For example, in northern elephant seals, males compete for females departing from harems. Further, dominant males can interrupt mating pairs, which can lead to severe injuries and even death for females (Le Boeuf and Peterson 1969; Le Boeuf and Mesnick 1991). Male black-headed gulls guard territories and can be physically aggressive to visiting females (Bastock et al. 1953). Overall, female harm caused by male-male competition may be widespread across animal taxa, and more studies should investigate its frequency as well as the evolutionary consequences for females.

In this study, we documented eight types of injuries females received (Fig. 3). All of the types were found at least once in females from the mixed-sex treatment while only four types were found in the female-only treatment (Fig. 2). Why did injuries occur at all in the female-only treatment? It is possible that females going into the experiment had minor injuries that we were only able to later see under the microscope. It is also possible that injuries like wing tears or wing fragmentation occurred due to aging and when females flew into cactus spines or the side of the container. Winged insects, such as the yellow dung fly, can accumulate wing injuries as they age (Burkhard et al. 2002). The number of wing tears documented in the mixed-sex treatment (17 cases) was almost three times more than those in the female-only treatment (6 cases), while the number of broken spines was 8 times more (16 compared to 2 cases), findings which strongly suggests that injuries incurred in the mixed-sex treatment were not merely due to aging or accidents. Pilot work on injuries in males revealed that 81% males living in mixed-sex group were injured compared to 27% males living alone (Raina et al. *unpublished manuscript*). This work has also suggested that male *N. femorata* commonly receive wing injuries from male-male competition, though antennal injuries are rare. In males, like in females, injuries to the ventral side of the body are rare. Interestingly, males appear to experience substantial tarsal injuries (Raina et al. *unpublished manuscript)*, while we found no tarsal injuries in females.

It is essential to understand the possible causes of female physical injuries because injuries might lead to fitness consequences via impaired locomotory ability (Emberts et al. 2021), increased energetic expenditure (von Wyschetzki et al. 2016; Emberts et al. 2021), and shortened lifespan (White and Bell 1993). In winged insects, flying ability can be essential as it allows individuals to avoid predators, find resources, and seek mates (Engel et al. 2013). Here, we found that wing injury was a common type of injury in both treatments (9 cases in female-only treatment and 21 cases in mixed-sex treatment). Nineteen out of 37 injured females exhibited wing tear or wing fragmentation, which composed 30 out of 64 cases of injury. Thus, injuries from males may hamper the ability of females to fly well, with a myriad of downstream consequences. In addition, 3 females in the mixed-sex treatment experienced antennal injuries which include missing setae and broken antennae, which might affect their ability to receive odor cues, with negative consequences for finding food and mates (Elgar et al. 2018).

While we documented all visible external injuries, it is possible that male *N. femorata* may cause damage to females that is difficult to observe. For example, 9 out of 13 females that were witnessed receiving at least one hindleg squeeze from males did not have observable external injuries. These interactions may result in internal injuries such as muscle damage or internal organ compression to the female, which are not readily visible. It is notable that males in leaf-footed bugs have a sclerotized genital clasper that can be used to grasp the female once mating has begun (E.V. Greenway *personal observation*). The function of their genital clasper and whether it can cause internal damage to the females are unknown. However, females appear to be able to quickly terminate copulation in *N. femorata* and walk away, which might not be the case for other leaf-footed bugs. In other insect species such as the bruchid beetle *Callosobruchus maculatus*, males possess sclerotized genitalia that are known to injure the female reproductive track during mating (Crudgington and Siva-Jothy 2000; Eady et al. 2007). Our design compared injuries in females in female-only groups versus females in mixed-sex groups. Thus, this design cannot exclude the possibility that simply living with even one male might increase female injury.

The second major finding of our study is that larger females were more likely to be injured. Interestingly, larger female *N. femorata* have thicker, more injury-resistant cuticle than smaller females (Greenway et al. 2024). So, we would expect larger females to show fewer injuries than smaller females if attacked at the same rate. It is possible that larger females behave differently than smaller females, and these behavioral differences have resulted in the pattern observed. Further, males may fight more vigorously over large females. Male mate choice is common across species (Bonduriansky 2001), and across taxa males frequently prefer larger females because size can be associated with fecundity (Verrell 1995; Bonduriansky 2001; Zahradnik et al. 2008; Pack et al. 2009; Edward and Chapman 2011). Gillespie et al. (2014) showed that males of *N. femorata* do indeed prefer to mate with larger, and thus, more fecund females (Gillespie et al. 2014), and male-male competition is more likely to be incited when females are present (Procter et al. 2012). However, to our knowledge, little research has examined whether males are more willing to fight over large females.

We did not find a correlation between female injury and reproductive output (Figure S2), which might be because we only measured female reproductive output over a very short period of time instead of their entire lifetime. Yet, studies on *Rhodinus prolixus* (Hemiptera: Reduviidae) showed that response to injuries can be activated within 6 h after being injured, starting with changing and spreading of the surrounding cells of the injured site (Wigglesworth 1937). Immune response can be costly (Bascuñán-García et al. 2010) and therefore, insects would presumably allocate more resources into immune response for wound healing instead of reproduction after getting injured. The fact that we did not see an effect of injury on offspring production suggests that the short-term immune response, if present, might not impose an immediate, measurable change in reproductive physiology. Many studies that have measured long-term female reproduction found a negative effect of male harassment, resulting in reduced lifetime reproductive success (Gay et al. 2009; Rossi et al. 2010; Fan et al. 2015).

In conclusion, the results support our hypotheses that females living with multiple males receive more external injuries and that larger females are more likely to be injured. In this case, the potential benefits of being around fighting males, such as obtaining relatively high-quality sperm, might not necessarily outweigh the cost of being harmed, especially for larger females. Female harm experienced via male-male competition may be common in nature, but it has rarely been examined. Future research should look more closely into when and how females are harmed by fighting males.

## Supplementary Material

araf068_suppl_Supplementary_Tables_S1-S2_Figures_S1-S2

araf068_suppl_Supplementary_Video_S2

araf068_suppl_Supplementary_Video_S1

## Data Availability

Analyses reported in this article can be reproduced using the data provided by Li and Miller (2025).
