# Supplementary material for: Living with males leads to female physical injury in the leaf-footed cactus bug
Source: Behav Ecol. 2025 Jun 11;36(4):araf068. doi: 10.1093/beheco/araf068 (PMC12207884; doi:10.1093/beheco/araf068)
Supplement: araf068_suppl_Supplementary_Tables_S1-S2_Figures_S1-S2 [file araf068_suppl_supplementary_tables_s1-s2_figures_s1-s2.docx]

**Supplementary Materials**

Living with males leads to female physical injury in the leaf-footed cactus bug

Yichen Li^1,2^ & Christine W. Miller^1,2*^

^1^Entomology & Nematology Department, University of Florida, 1881 Natural Area Drive, Gainesville, FL 32611, USA

^2^Current address: University of Cambridge, Department of Zoology, Downing Street, Cambridge CB2 3EJ

*Corresponding author: Christine W. Miller, University of Cambridge, Department of Zoology, Downing Street, Cambridge CB2 3EJ. Email: [cwm30@cam.ac.uk.](mailto:cwm30@cam.ac.uk.)

**Figure S1.** Female body size is positively correlated with female reproductive output.

**
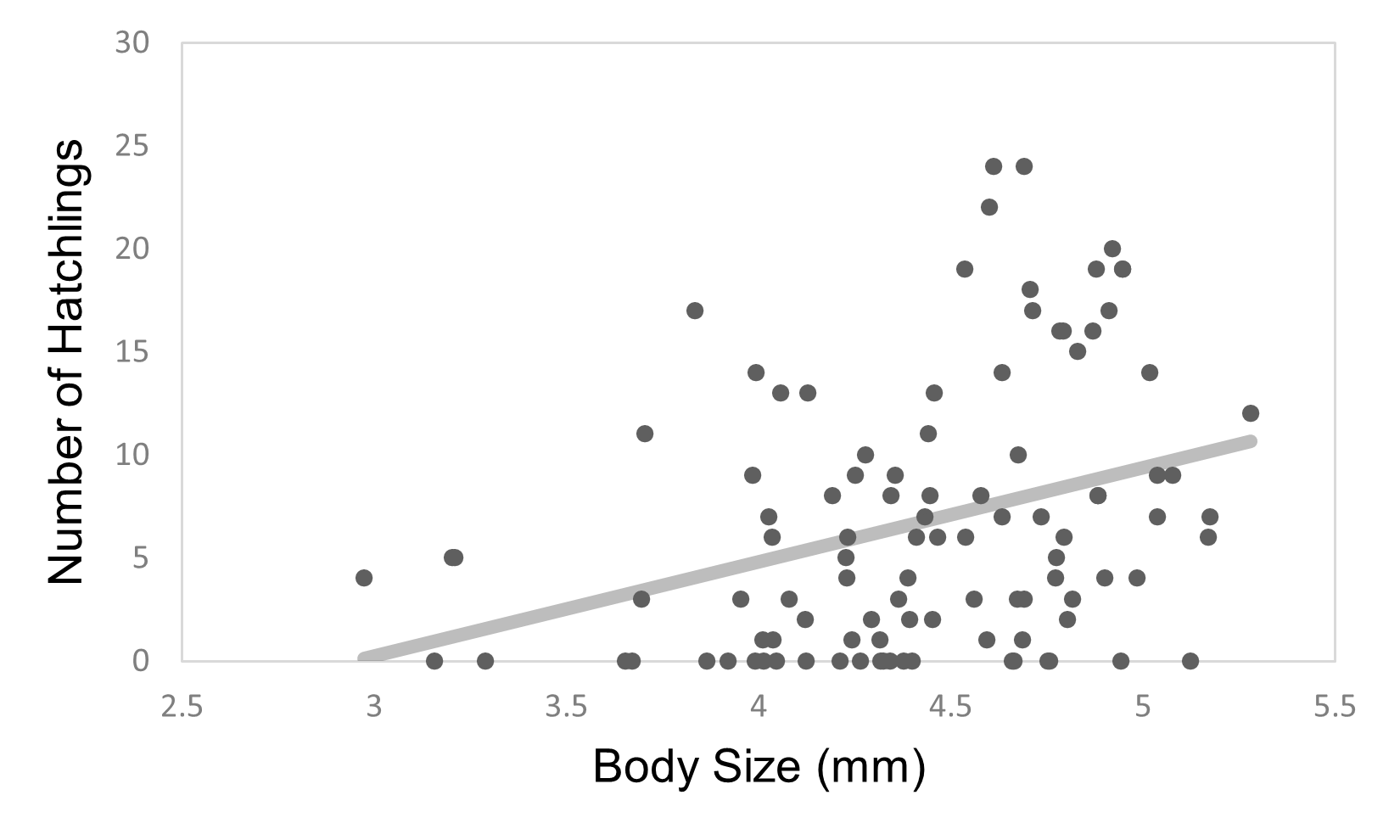
**

**Figure S2.** We did not find an association between female injury and the number of hatchlings produced.


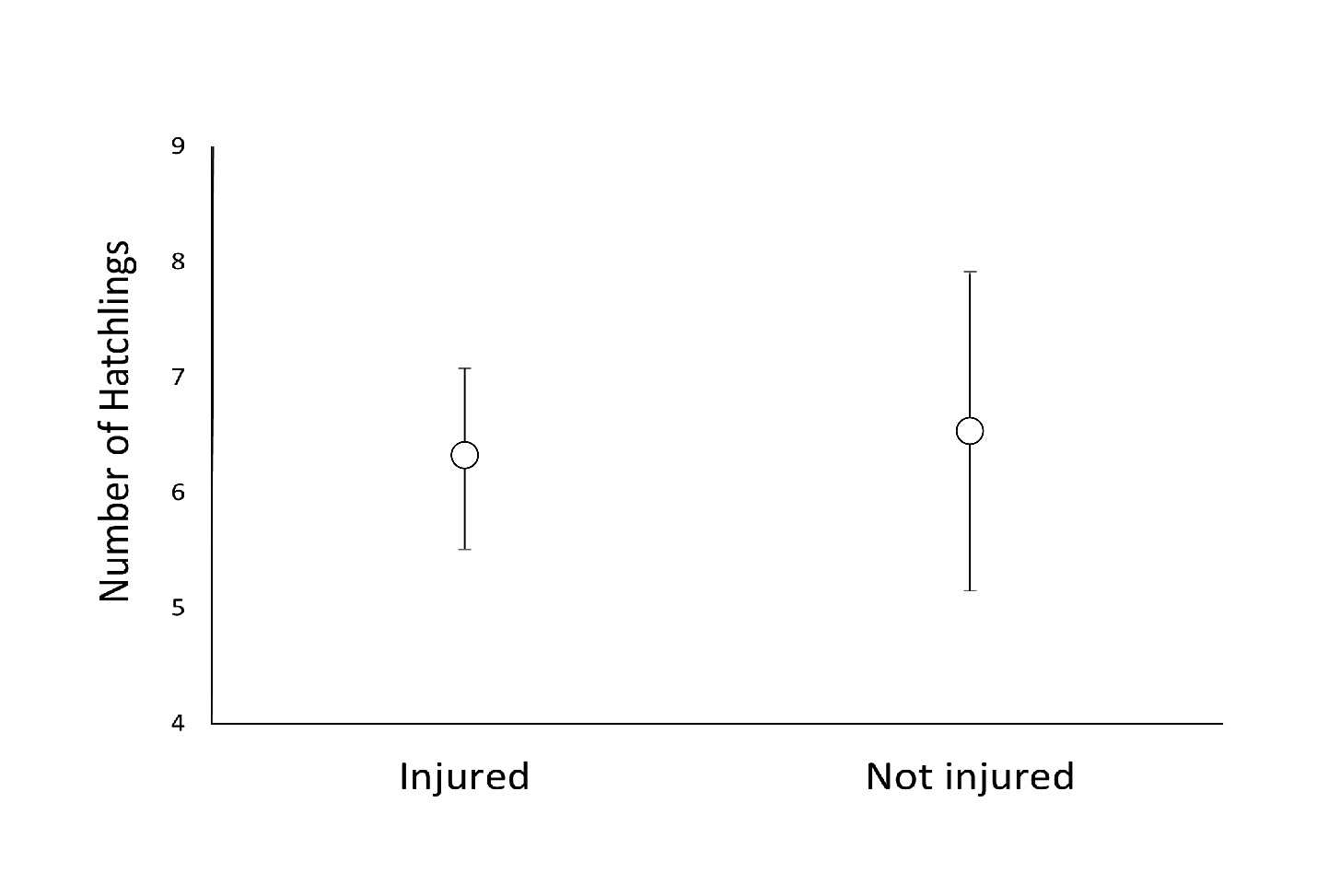


**Table S1.** Ethogram of *Narnia femorata* social behaviors (modified from Nolen et al. 2017). This table highlights the behaviors that are frequently observed in social groups with more than one male.

| **Behavior** | | **Description** |
| --- | --- | --- |
| Approach | | When one insect moves slowly toward another. This is only recorded if the insects are within 3cm from tip to tip. |
| Leg display | | When one insect raises one or both hindlegs in the direction of another insect without physical contact. |
| Charge | | When one insect, usually a male, moves very rapidly toward another individual and typically has one hindleg extends toward the other individual at the end. |
| Contact | | When one insect contacts another without the other behaviors described below such as mounting or kick. Examples: antennal contact or standing adjacent. |
| Mount | | When one insect climbs on back of another individual or have legs overlap another’s back and stays in such position for at least 3 seconds instead of just walking by. |
| Kick | | When one strikes another individual using one or both hindlegs. |
| Squeeze | “Bear-hug” squeeze | When one insect wraps around another’s body and squeezes with both hindlegs simultaneously. |
|  | “Bicep-curl” squeeze | When one wraps a part of another insect’s body using only one of their hindlegs, squeezing together the tibia and femur. |

**Table S2.** Injury measurements.

| **Treatment** | **General Body Location** | **Injury Type** | **Sample ID** | **Length (mm)** | **Area (mm2)** | **Which Wing** |
| --- | --- | --- | --- | --- | --- | --- |
| Female-only | Wing injury | Wing fragmentation | F65 |  | 0.032 | Hindwing |
|  |  |  | F80 |  | 0.083 | Forewing |
|  |  |  | F227 |  | 0.099 | Forewing |
|  |  | Wing tear | F70 | 0.329 |  | Forewing |
|  |  |  |  | 0.085 |  | Forewing |
|  |  |  | F80 | 0.388 |  | Forewing |
|  |  |  |  | 0.292 |  | Forewing |
|  |  |  |  | 0.077 |  | Forewing |
|  |  |  | F135 | 0.754 |  | Forewing |
| Mixed-sex | Antennal injury | Broken antennae | F37 | 5.843 |  |  |
|  |  | Missing setae | F209 | 0.624 |  |  |
|  | Leg injury | Exoskeletal puncture | F122 |  | 0.059 |  |
|  |  |  | F112 |  | 0.149 |  |
|  | Wing injury | Wing fragmentation | F73 |  | 0.392 | Forewing |
|  |  |  | F73 |  | 2.384 | Forewing |
|  |  |  | F46 |  | 1.125 | Forewing |
|  |  |  | F112 |  | 0.083 | Hindwing |
|  |  | Wing tear | F73 | 0.29 |  | Hindwing |
|  |  |  |  | 0.205 |  | Hindwing |
|  |  |  |  | 0.183 |  | Hindwing |
|  |  |  | F120 | 0.261 |  | Forewing |
|  |  |  | F109 | 0.119 |  | Forewing |
|  |  |  | F35 | 2.105 |  | Forewing |
|  |  |  | F61 | 1.635 |  | Forewing |
|  |  |  | F46 | 3.296 |  | Forewing |
|  |  |  |  | 0.973 |  | Forewing |
|  |  |  |  | 0.445 |  | Forewing |
|  |  |  | F181 | 0.919 |  | Forewing |
|  |  |  |  | 0.682 |  | Forewing |
|  |  |  | F131 | 0.615 |  | Forewing |
|  |  |  |  | 0.196 |  | Forewing |
|  |  |  |  | 1.287 |  | Forewing |
|  |  |  | F205 | 0.223 |  | Forewing |
|  |  |  | F214 | 1.093 |  | Forewing |

**Video S1**: Example of a male squeeze attacking a female that were not engaged in copulation. In this example, the female is experiencing a bicep-curl squeeze on her left antenna. Female insect is painted in red on pronotum and male is painted in green on pronotum.

**Video S2**: Example of mating pair harassment. In this example, one male is approaching, interrupting, and engaging in fights with a male that is trying to mate with the female. Female insect is painted in red on pronotum and male is painted in green on pronotum.
